# Prognostic significance of new onset ascites in patients with pancreatic cancer

**DOI:** 10.1186/1477-7819-4-16

**Published:** 2006-03-28

**Authors:** Emmanuel E Zervos, Dana Osborne, Brian A Boe, German Luzardo, Steven B Goldin, Alexander S Rosemurgy

**Affiliations:** 1 Digestive Disorders Center, Tampa General Hospital, Tampa, Florida, USA; 2 James A. Haley Veterans Administration Hospital, Tampa, Florida, USA; 3 Department of Surgery, University of South Florida, USA

## Abstract

**Background:**

The purpose of this study was to determine risk factors for development of malignant ascites and its prognostic significance in patients with pancreatic cancer.

**Methods:**

A prospective database was queried to identify patients with pancreatic cancer who develop ascites. Stage at presentation, size, and location of primary tumor, treatment received and length of survival after onset of ascites were determined.

**Results:**

A total of 15 patients were identified. Of which 4 patients (1 stage II, 3 stage III) underwent pancreaticoduodenectomy and manifested with ascites 2, 3, 24 and 47 months after surgery (tumor size 2.9 ± 1.32 cm). All but one of the remaining 11 patients (tumor size 4.4 ± 3.38 cm) presented with metastatic disease, and all developed malignant ascites 9 months after diagnosis, dying 2 months later. Resected patients lived longer before the onset of ascites, but not after.

**Conclusion:**

Once diagnosed, ascites in pancreatic cancer patients heralds imminent death. Limited survival should be considered when determining the aggressiveness of further intervention.

## Background

Pancreatic cancer is the 5^th ^most common gastrointestinal (GI) malignancy but the third most common cause of death among all GI cancers [1]. Almost all patients with pancreatic cancer ultimately die of it, usually within 6 months of diagnosis [2]. The vast majority of these patients are ineligible for surgical therapy at the time of diagnosis due to metastatic spread or local invasion and death usually occurs by inanition rather than gross tumor burden. Aggressive chemotherapeutic regimens are generally highly toxic and prolong life by weeks, rather than months [3]. Most patients with unresectable pancreatic cancer die of their disease before completing a standard course of therapy.

Common manifestations of end stage pancreatic cancer include: gastric outlet obstruction due to tumor ingrowth to the duodenum, cachexia, deep venous thrombosis (Trousseau's phenomenon), anasarca and ascites. Ascites manifests in only 20% of pancreatic cancer patients and its cause is multifactorial [4]. It can occur due to obstruction of diaphragmatic lymphatics, increased production of exudate by the tumor itself and production of osmotically active peptides that alter vascular permeability to favor ascites formation [5]. In most series, over half of the time, cancer cells can not be identified in a paracentesis sample [4,6]. As such, in this setting, the ascites may not be malignant in the purest sense however; it is undoubtedly due to the cancer even if cancer cells cannot be found in the fluid. Other authors have suggested methods other than cytology to determine the nature of ascites such as ascites/serum albumin gradients or presence of various substances such as telemorase or human gonadotropin-β in the ascites [7-9]. We have noted that regardless of whether ascites can be proven to be malignant cytologically, it is usually the final manifestation of a uniformly fatal diagnosis.

Malignant ascites can be managed for extended periods in patients with other types of cancer such as those of gynecologic, gastric or colonic origin [10]. We have noted, however, after treating multiple patients with both resected and non-resected pancreatic cancer on various protocols at our institution that regardless of the type of intervention employed, the new onset of ascites in these patients usually heralded imminent demise. The purpose of this study, therefore, was to examine this phenomenon and determine the exact prognostic implications of new onset ascites to better guide subsequent treatment and to counsel patients more effectively. We hypothesized that new onset ascites in patients with pancreatic cancer would be associated with those factors commonly attributed to the final stages of the disease and that death would soon follow its manifestation.

## Patients and methods

A prospective database involving all patients treated on protocols for pancreatic cancer at our institution was begun in 2001. All patients were entered into this database with Institutional Review Board (IRB) approval after giving informed consent. The database chronicles each patient's course from the time of enrollment into a protocol to the time of death, regardless of whether they complete the protocol. Patients who presented with, or developed ascites after having been diagnosed with pancreatic cancer were included in this analysis. Chart review was undertaken to gain basic demographic data including patient age, gender, tumor characteristics such as size, and location. Stage and tumor marker levels were determined at three different time points: presentation, onset of ascites and just prior to death.

After identifying all patients with ascites, patients were divided into two groups: those having undergone resection and those managed non-operatively. The type of treatment employed for both the primary tumor and the ascites was noted. The incidence of malignant cells in the ascites was noted as was the method by which the ascites was diagnosed. Overall survival, ascites free interval and survival with ascites were determined for the entire cohort. Survival after therapeutic intervention was also noted after separating the patients into those undergoing no treatment, diuretics alone, paracentesis alone, diuretics and paracentesis or peritoneovenous shunting. Double valved Denver^® ^(Denver Biomaterials, Denver Colorado) peritoneovenous shunts were employed in all patients undergoing this type of treatment. In cases where a patient's status was unknown, attempts were made to contact the patient or their family. All data is current to March of 2005.

The database was queried to determine whether any specific predictors of risk of developing malignant ascites could be gleaned. Linear regression was utilized to determine whether tumor size, lymph node status, margin status (if resected), grade, stage at presentation, tumor location, stage at onset of ascites, presence of neural or lymphovascular invasion, or type of therapy (chemotherapy *v*. chemoradiation) would predict the onset of ascites.

Where appropriate, tumor size and survival characteristics were compared using a two tailed Student's T-test. Significance was accepted with 95% probability.

## Results

### Demographics and basic tumor characteristics

A total of 120 patients were enrolled in protocols at our institution between 2001 and late 2004. Of these 120 patients, 15 were identified who developed ascites after the diagnosis of pancreatic cancer. All but two were men and average age was consistent with that which is commonly observed in this disease process. Almost all tumors were located in the head of the gland, one patient presenting with intermittent jaundice, an elevated carbohydrate antigen 19-9 (CA 19-9) level and no radiographic evidence of disease was diagnosed with peritoneal carcinomatosis and ascites at the time of celiotomy; his primary tumor was occult. Four patients underwent resection of their tumors and although their tumors were almost 1.5 cm smaller at presentation than those patients not undergoing resection, this difference was not statistically significant. Ascites was most commonly diagnosed by physical examination followed by radiographically and then as an incidental finding at celiotomy. In these patients, ascites manifested between the time of their preoperative computed tomography (CT) scan suggesting resectability and the time of their exploration and none were resected. Demographics for the entire group of patients are depicted in Table 1.

**Table 1:** Patient Demographics

	**Number of patients**
**Age at diagnosis**	60 ± 10.4 years
**% Male**	87%
**Location of Tumor**	
Head	13
Body	0
Tail	1
Unknown	1
**Average size of tumor (cm)**	
Overall	4.2 ± 3.04 cm
Resected	2.9 ± 1.32 cm
Unresectable	4.4 ± 3.38 cm
**Presentation**	
Resected	4
Locally Advanced	6
Metastatic	5
**Ascites Diagnosed**	
At celiotomy	2
Physical exam	10
Radiographically	3

### Cytology

**Figure 1:**
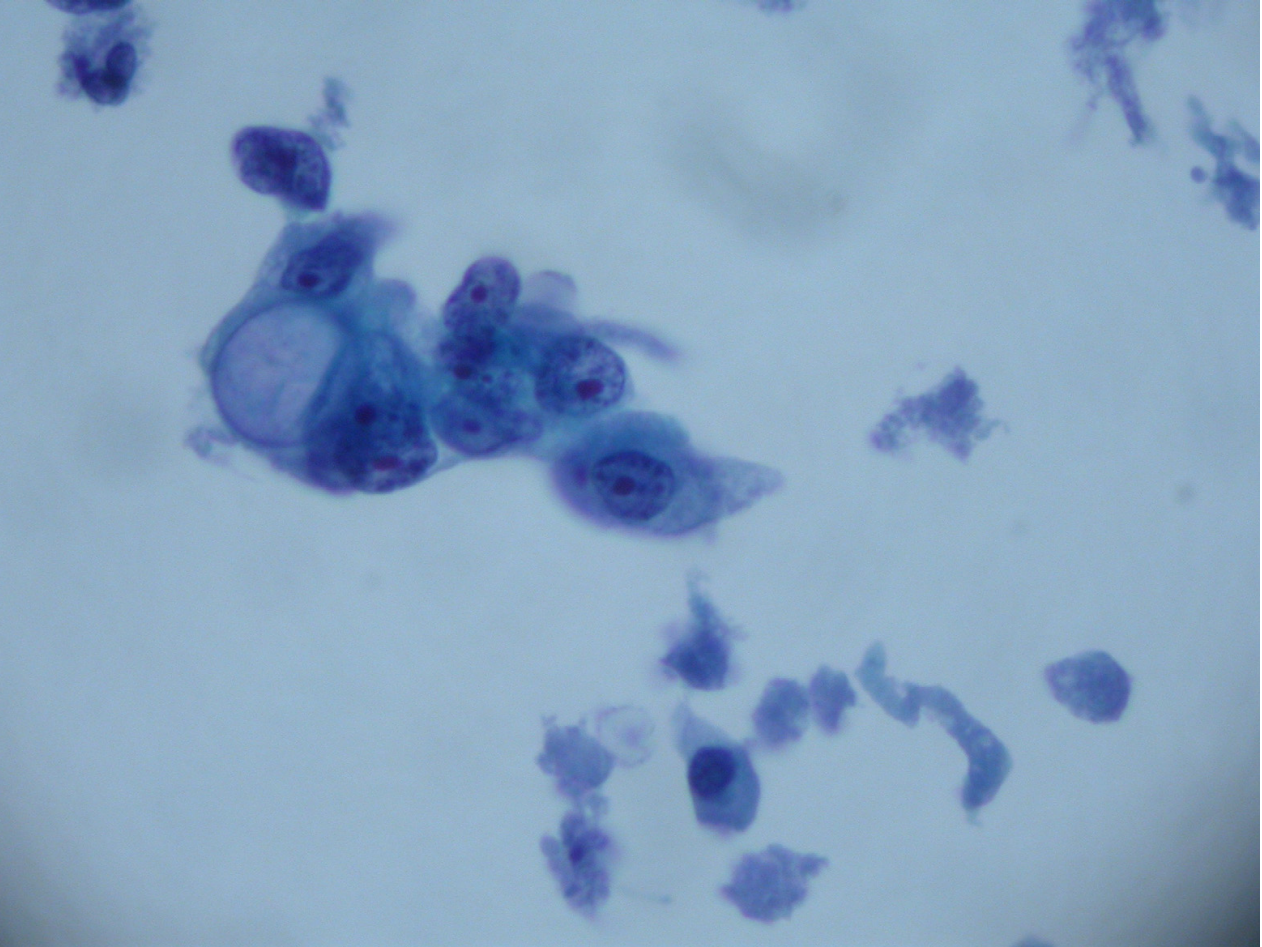
Photomicrograph with cytologically positive specimen in a patient with pancreatic cancer and new onset ascites (hematoxylin and eosin stain, ×1000).

All patients underwent paracentesis or intraoperative collection of fluid for cytologic analysis. Only 5 of the 15 patients in this study had malignant cells in their ascites. Figure 1 is a representative photomicrograph of a positive cytologic preparation in one such patient.

### Survival

Survival for all patients, those undergoing resection and those receiving non-operative management of their cancer is summarized in Table 2. Overall survival was just less than one year for the group as a whole. Survival in resected patients was near significantly longer (p = 0.06) than non-resected patients. Ascites free survival was significantly longer in patients undergoing resection but once ascites became manifest, survival was no different in patients who had undergone resection than those not undergoing resection. Once ascites developed, patients treated non-operatively lived about 7 weeks longer than those that developed ascites after pancreaticoduodenectomy. Overall survival after ascites developed was slightly over 2 months.

**Table 2:** Survival in months

	**Overall**	**Resected**	**Unresected**	**p value**
**n**	15	4	11	
**From diagnosis**	11.2 ± 12.10	20.8 ± 20.22	7.7 ± 5.65	0.06
**Ascites free**	9.0 ± 12.06	20 ± 20.32	5.1 ± 3.86	0.02
**With ascites**	2.2 ± 2.56	0.8 ± 0.43	2.6 ± 2.85	0.24

### Survival based on treatment rendered for ascites

Patients managed with diuretics alone or peritoneovenous shunts seemed to do better in this group than those receiving no treatment or paracentesis with or without diuretics although none of these intervals were statistically different. Table 3 chronicles outcome based on treatment method.

**Table 3:** Survival by Treatment Method

	**None**	**Diuretics**	**Paracentesis**	**Both**	**PV shunt**
**N**	3	3	3	3	3
**Survival (months)**	1.2 ± 1.16	2.7 ± 2.61	1.2 ± .80	1 ± .73	4.7 ± 4.63

### Stage and tumor markers

Patients were evenly distributed between stages at the time of presentation but quickly progressed to Stage IV disease at the time their ascites was discovered. This includes those patients who were not automatically staged as metastatic because of positive fluid cytology. Ascites rarely occurred in patients who were not advanced stage.

Tumor markers were higher at diagnosis than at the onset of ascites reflecting again the advanced nature of the disease at the time it was discovered. At presentation of ascites, tumor markers were lower, but quickly reached their highest point at those levels drawn just before each patient's death. Wide variations in levels of tumor markers at each interval resulted in no statistical difference in CA 19-9 at various time points.

### Multivariate analysis

Linear regression revealed no characteristics to predict the onset of ascites. Tumor size approached but given the size of the overall database, did not reach statistical significance. Location, lymph node status, histologic grade, and type of or utilization of chemotherapy or chemoradiotherapy neither predicted nor prevented new onset ascites. Presence of unexplained free fluid at the time of celiotomy always predicted eventual onset of ascites, but this was not categorized as "ascites" in the database and, as such, was not predictive.

## Discussion

Pancreatic cancer remains a diagnostic and therapeutic challenge. No effective medical therapies have emerged, despite rapid advancements in targeted chemotherapeutic agents. Patients with pancreatic cancer continue to die with relatively low tumor burden when compared to other cancer types. Ascites, whether proven to be malignant or not, is a harbinger of the final stages of pancreatic cancer. It occurs relatively infrequently, in part because many patients do not survive long enough to manifest with it. This is the first study that documents a commonly observed clinical phenomenon; the appearance of ascites in pancreatic cancer is associated with minimal remaining life expectancy, regardless of prior intervention for the treatment of the primary tumor and regardless of how the ascites is managed.

In this study, we have looked at 15 patients obtained from a common database composed of patients enrolled in various adjuvant and palliative protocols for pancreatic cancer at our institution. Most patients had tumors located in the head of their gland, and over two thirds were unresectable. As one would expect, the ascites free interval was longer in the four patients undergoing complete resection of their tumor, but once the tumors recurred, as almost all do, life expectancy with ascites was less than one month. There were no predictive indicators of who would develop ascites based on findings at initial presentation. Patients who were not resected did slightly better in this regard in that they survived slightly longer after the appearance of ascites, and this is probably a manifestation of the survival benefit, albeit minimal, of ongoing cytotoxic chemotherapy. Other authors have shown median survival of approximately the same length once ascites manifests [11]. Nonetheless, the salutary effects of systemic chemotherapy were not great enough to produce a meaningful difference in survival with ascites between resected patients who had completed their therapy months earlier and non resected patients who were undergoing therapy when the ascites appeared.

Tumor markers did not correlate with onset of ascites, and this is not surprising given that only one in three patients actually had tumor cells in their ascites fluid; perhaps this is the most important observation that can be gleaned from this study. Cytology does not appear to be important – the mere *presence *of ascites is what really matters. Large tumors shed tumors cells, but smaller tumor secrete vasoactive peptides that change osmotic gradients and cause ascites; this was probably the case in patients with ascites and no carcinomatosis or with radiologically occult primaries. Most patients never came to surgery to document the presence or absence of carcinomatosis; we presume it was present but cannot say for sure. Regardless of whether carcinomatosis could be documented, ascites, in and of itself, was a uniformly poor prognostic sign. This statement is supported by the wealth of data regarding the grave prognostic implications of positive peritoneal washings in patients with pancreatic cancer and radiologically resectable tumors [12-15]. At our institution, routine washings of the peritoneum are not obtained. First and foremost, resection is pursued based upon preoperative imaging studies and intraoperative findings since it offers the best survival advantage. As a result of this analysis, we now regard the presence of unexplained intraperitoneal fluid at the time of celiotomy as an absolute contraindication to resection.

## Conclusion

Based on our clinical impression and now, this study, when we observe it, we seriously weigh the utility of continuing chemotherapy with toxic side effects. Our focus shifts towards palliation and supportive care and, although peritoneovenous shunts do not prolong life, we consider them supportive care in this patient group. Anything that keeps patients out of the hospital and reasonably palliated can only serve to enhance the short time left in the course of their illness.

## Competing interests

The author(s) declare that they have no competing interests.

## Authors' contributions

EZ was responsible for acquisition of substantial data, drafting of manuscript, and study design. **DO **was responsible for managing database, statistical analysis, data aquisition and study design. **BB **maintained database and assisted in drafting the manuscript. **GL **was responsible for identifying patients and review of patient records. **SG **was involved in coordinating study and design. **AR **was responsible for study conception and design.

All authors read and approved the final version of the manuscript.

**Table 4:** Stage and tumor markers

	**At diagnosis**	**At ascites**	**At death**
**AJCC Stage**			
**II**	5	1	0
**III**	2	3	2
**IV**	8	11	13
**CA 19-9**	**1532 ± 2751**	**707 ± 761**	**2948 ± 5531**
